# Supplementary material for: Causal Relationship between Aging and Anorexia Nervosa: A White-Matter-Microstructure-Mediated Mendelian Randomization Analysis
Source: Biomedicines. 2024 Aug 16;12(8):1874. doi: 10.3390/biomedicines12081874 (PMC11351342; doi:10.3390/biomedicines12081874)
Supplement: Supplementary file 1 [file biomedicines-12-01874-s001.zip › Supplementary Figure/Figure S8/Leaveoneout plot of MR analysis for TL to ubm-b-1779.pdf]

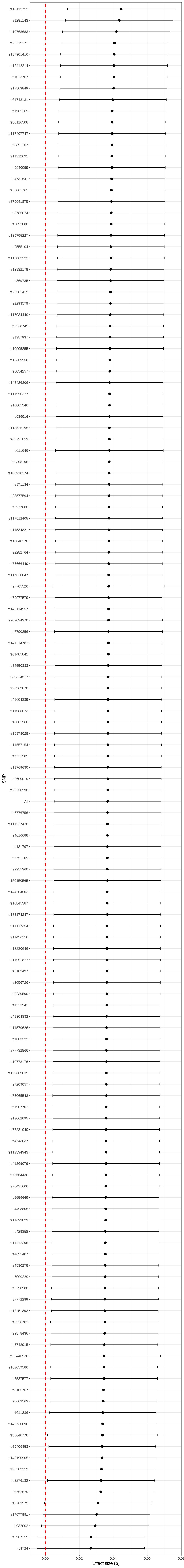
SNP
rs10112752
rs1291143
rs10768683
rs76219171
rs137901416
rs12412214
rs1023767
rs17803849
rs61748181
rs1985369
rs80116508
rs117407747
rs3891167
rs11212631
rs9940099
rs4731541
rs56061761
rs376641875
rs3785074
rs3093888
rs139795227
rs2555104
rs116863223
rs12932179
rs869785
rs73581419
rs2293579
rs117034449
rs2538745
rs1957937
rs10905255
rs12369950
rs6054257
rs142426306
rs111950327
rs10805346
rs939916
rs113525195
rs66731853
rs611646
rs9398196
rs188918174
rs871134
rs28577594
rs2977608
rs117512405
rs11584821
rs10840270
rs2282764
rs76666449
rs117630647
rs7705526
rs79977579
rs145114957
rs202034370
rs7790856
rs141214782
rs61405042
rs34550383
rs80324517
rs28363070
rs45604339
rs11085072
rs6881568
rs16978028
rs11557154
rs7221585
rs11769630
rs9600019
rs73730598
All
rs6776756
rs111527438
rs4616688
rs131797
rs6751209
rs9955360
rs150150565
rs144204502
rs10845387
rs185174247
rs11117354
rs11426156
rs13230646
rs11991877
rs8102497
rs2056726
rs2230590
rs1332941
rs41304832
rs11579626
rs1003322
rs77732866
rs10773176
rs139669835
rs7209057
rs76065543
rs1907702
rs13062095
rs77231040
rs4743037
rs112394943
rs41269078
rs75664430
rs78491606
rs6659669
rs4498805
rs11699829
rs429358
rs11412296
rs4695407
rs4530278
rs7099229
rs6790988
rs7772289
rs12451892
rs6536702
rs9878436
rs5742915
rs35446936
rs182059586
rs6587577
rs8105767
rs6669563
rs1611236
rs142730696
rs35640778
rs59409453
rs143190905
rs28502153
rs2276182
rs762679
rs2763979
rs17677991
rs932002
rs2967355
rs4724
0.00
0.02
0.04
0.06
Effect size (b)
